# Synergistic Enhancement of Antitumor Effects by Combining Abemaciclib with Desipramine

**DOI:** 10.3390/ijms25137407

**Published:** 2024-07-05

**Authors:** Yan Li, Yeojin Sung, Young Eun Choi, Yongdoo Choi, Sung-Ho Goh

**Affiliations:** 1 Division of Technology Convergence, National Cancer Center, 323 Ilsan-ro, Goyang 10408, Gyeonggi-Do, Republic of Korea; liyan81@ncc.re.kr; 2 Division of Cancer Biology, National Cancer Center, 323 Ilsan-ro, Goyang 10408, Gyeonggi-Do, Republic of Korea; yjsung@ncc.re.kr (Y.S.); 75162@ncc.re.kr (Y.E.C.)

**Keywords:** abemaciclib, tricyclic antidepressant, combined therapy, in vivo

## Abstract

Cyclin-dependent kinase 4 and 6 (CDK4/6) inhibitors, including abemaciclib, have been approved for the treatment of hormone receptor-positive, human epidermal growth factor receptor 2 (HER2)-negative advanced, and metastatic breast cancer. Despite the high therapeutic efficacy of CDK4/6 inhibitors, they are associated with various adverse effects, including potentially fatal interstitial lung disease. Therefore, a combination of CDK4/6 inhibitors with letrozole or fulvestrant has been attempted but has demonstrated limitations in reducing adverse effects, highlighting the need to develop new combination therapies. This study proposes a combination strategy using CDK4/6 inhibitors and tricyclic antidepressants to enhance the therapeutic outcomes of these inhibitors while reducing their side effects. The therapeutic efficacies of abemaciclib and desipramine were tested in different cancer cell lines (H460, MCF7, and HCT-116). The antitumor effects of the combined abemaciclib and desipramine treatment were evaluated in a xenograft colon tumor model. In vitro cell studies have shown the synergistic anticancer effects of combination therapy in the HCT-116 cell line. The combination treatment significantly reduced tumor size compared with control or single treatment without causing apparent toxicity to normal tissues. Although additional in vivo studies are necessary, this study suggests that the combination therapy of abemaciclib and desipramine may represent a novel therapeutic approach for treating solid tumors.

## 1. Introduction

In most cancer cells, the rate of cell proliferation is abnormally increased, often because of dysregulated cyclin-dependent kinase (CDK) activity [1,2]. CDKs are enzymes essential for the concerted regulation of cyclin activities throughout the cell cycle and eventually for the progression of cell proliferation [3]. Therefore, tumor suppression by the inhibition of abnormal CDK activity has emerged as a potential therapeutic strategy for various cancers.

Cyclin D1 determines the progression from the G1 phase to the S phase of the cell cycle, and CDK4/6 phosphorylates and activates cyclin D1 to regulate the cell cycle [4]. CDK4/6 inhibitors block the formation of the CDK4/6–cyclin D1 protein complex, thereby halting cell division [2]. Three CDK4/6 inhibitors, namely palbociclib, ribociclib, and abemaciclib, have been approved for the treatment of hormone receptor (HR)-positive, HER2-negative advanced, or metastatic breast cancers [4,5]. Among these inhibitors, abemaciclib has a broader spectrum of potential uses beyond breast cancer, and its ability to cross the blood–brain barrier and its efficacy in other cancer types are being explored in clinical trials, making it a relevant choice for various cancers [6].

Despite the high therapeutic efficacy of CDK4/6 inhibitors, they exhibit various adverse effects. Although the risk of most side effects is low (grade 1 or 2), more severe effects (grade 3 or 4) such as gastrointestinal disturbances, neutropenia, and leukopenia, which can lead to infections, are frequently observed [7]. Severe and potentially fatal interstitial lung disease has also been identified [8]. Thus, either discontinuing the use of CDK4/6 inhibitors or reducing their dosage is necessary to mitigate adverse effects [7,9].

Several clinical trials have explored combination therapies using CDK4/6 inhibitors [10]. For example, combination therapy with letrozole, an estrogen-converting inhibitor, or fulvestrant, an estrogen blocker, which are drugs used in hormonally positive (HR+) breast cancer treatment, has been tested with palbociclib, ribociclib, and abemaciclib [11,12,13]. However, these combination therapies have limitations in terms of reducing adverse effects, thus highlighting the need to develop new combination therapies [14].

Patients with cancer frequently experience depression because of pain associated with treatment, often necessitating antidepressant prescriptions that vary widely in dosage [15,16,17]. Antidepressants have side effects including insomnia, weight loss, dry mouth, and chest pain [18]. However, unlike the high-risk side effects of CDK4/6 inhibitors, dangerous side effects are not commonly associated with antidepressants [18]. Furthermore, recent studies investigating the survival rates of patients with cancer using antidepressants have shown that the use of antidepressants does not worsen survival rates. Moreover, tricyclic antidepressants (TCAs) significantly improve the survival rates of patients with lung cancer [15,16] and hepatocellular carcinoma [17].

**Figure 1:**
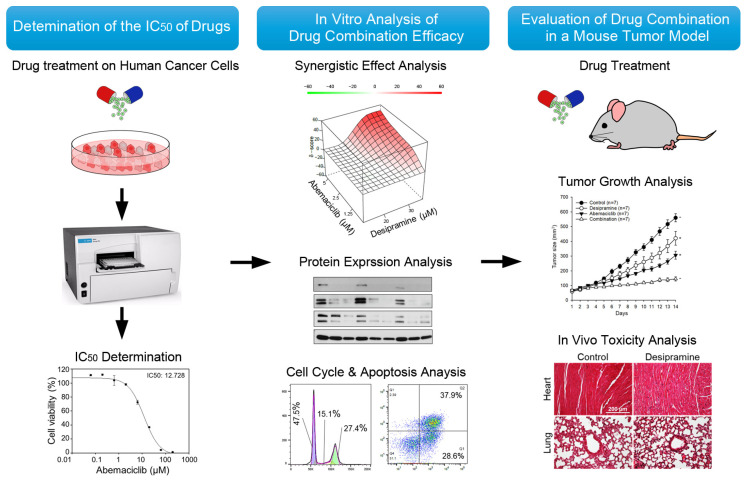
The workflow and study design for analyzing the efficacy of combination therapies.

Herein, we propose the use of a TCA, desipramine, as a combination drug to enhance the therapeutic outcomes of CDK4/6 inhibitors. We performed in vitro cytotoxicity tests using a combination of various concentrations of abemaciclib and desipramine (Figure 1). In addition, in vivo animal studies were conducted to evaluate the antitumor effect of the combination of abemaciclib and desipramine in a human colon cancer-bearing mouse tumor model. As a result, the combination treatment significantly reduced the tumor size compared with the control or single treatment without causing apparent toxicity to normal tissues. Our findings suggest that the combination of abemaciclib and desipramine could reduce side effects and enhance tumor suppression, thus presenting a promising therapeutic strategy.

## 2. Results

### 2.1. Combined Treatment of Abemaciclib with Desipramine in Various Cancer Cell Lines

Considering the broad application range of abemaciclib, we selected three types of cancer cell lines for in vitro cell studies, targeting cancers with high global incidence rates according to Global Cancer Statistics 2022: lung cancer (12.4%), breast cancer (11.6%), and colorectal cancer (9.6%) [19]. For breast cancer, we chose the MCF7 cell line, which models triple-negative breast cancer (TNBC), despite CDK4/6 inhibitors typically targeting HR+ breast cancer. TNBC lacks both the progesterone receptor and HER2 protein, making chemotherapy a priority. Additionally, we selected the NCI-H460 cell line to model non-small-cell lung cancer (NSCLC) and the HCT-116 cell line for colorectal cancer (CRC). We then analyzed the effects of combination therapy with desipramine in these cell lines.

**Figure 2:**
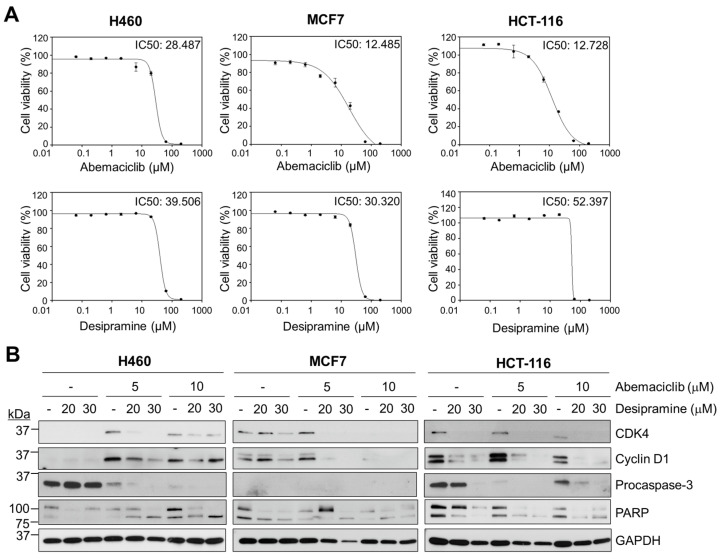
Effects of abemaciclib and desipramine on cell viability and protein levels related to cell cycle and cell death in various cancer cell lines. (**A**) Determination of IC_50_ of abemaciclib and desipramine treatment in H460, MCF7, and HCT-116 cells for 72 h (mean ± standard error). (**B**) Immunoblots of cell death-related protein and cell cycle-related proteins (CDK4 and cyclin D1) and apoptosis-related proteins (procaspase-3 and PARP) on abemaciclib, desipramine, and combination treatment in H460, MCF7, and HCT-116 cells for 72 h. GAPDH was used as the loading control. CDK4, cyclin-dependent kinase 4; PARP, poly (ADP-ribose) polymerase; GAPDH, glyceraldehyde 3-phosphate dehydrogenase.

To determine the anticancer effects of abemaciclib and desipramine, a CellTiterGlo assay was performed to analyze cell viability by measuring adenosine triphosphate levels in the cells. Although the IC_50_ of abemaciclib was similar between the breast cancer cell line MCF7 and the colon cancer cell line HCT-116 (12.5 vs. 12.7 µM), the NSCLC cell line, with H460 cells, showed a more than two-fold higher IC_50_ value (28.5 µM) than MCF7 or HCT-116 cells (Figure 2A, upper row). The IC_50_ values of desipramine were 39.5, 30.3, and 52.4 μM for H460, MCF7, and HCT-116 cancer cells, respectively (Figure 2A, bottom row). MCF7 breast cancer cells and HCT-116 colon cancer cells were more sensitive to abemaciclib treatment than H460 cells, although most cell lines were relatively insensitive to desipramine in terms of cell viability.

Thus, we examined changes in proteins related to the cell death process (poly (ADP-ribose) polymerase; PARP) and cell cycle progression (CDK4 and cyclin D1) in these cell lines following treatment with abemaciclib, desipramine, or their combination. In H460 cells, CDK4 and cyclin D1 protein levels were not reduced by single-drug treatment with abemaciclib (5 or 10 μM). Interestingly, the CDK4 level of H460 cells was rather increased upon abemaciclib treatment, as was reported previously [20,21], but decreased by combination with desipramine (20 μM). During apoptosis, active caspase-3 is known to cleave the precursor form of PARP. Caspase-3 itself exists as procaspase-3 and is activated through cleavage by caspase cascade, resulting in a reduction of its 32 kDa precursor form [22]. In H460 cells, neither reduction of procaspase-3 as activation signal nor induction of PARP cleavage was observed by desipramine alone. Meanwhile, caspase 3 was dramatically abolished with addition of abemaciclib. In accordance with caspase 3 decrement, cleaved PARP was increased at higher abemaciclib levels (10 μM). In MCF7 cells, a higher abemaciclib concentration (10 μM) was effective in abolishing CDK4 and cyclin D1 proteins. However, regardless of PARP cleavage, procaspase-3 expression was not detected in MCF7 cells, as was reported [23]. Interestingly, single-drug treatment of desipramine reduced CDK4 and cyclin D1 at higher concentration (30 μM) in MCF7. In HCT-116 cells, a higher abemaciclib concentration (10 μM) was more effective in reducing CDK4 or cyclin D1 than a lower abemaciclib concentration (5 μM), although they were not completely abolished, unlike MCF7 cells. Procaspase-3 level in HCT-116 cells was not affected by desipramine alone up to 20 μM but was abolished at 30 μM. Meanwhile, procaspase-3 was dramatically reduced by addition of abemaciclib, and PARP cleavage was also observed accordingly. However, as was observed in MCF7 cells, desipramine single-drug treatment of HCT-116 cells dramatically reduced CDK4 and cyclin D1 levels, although PARP cleavage was not increased. Thus, desipramine is a putative drug that arrests cell cycle progression without inducing cell death, though it is not known for having CDK4 inhibitor function.

Combining abemaciclib and desipramine enhanced PARP cleavage at a higher desipramine concentration (30 μM) in all three cell lines. However, this combination was not effective in reducing CDK4 and cyclin D1 levels in H460 cells but was only effective in such levels in HCT-116 cells. The effect on MCF7 cells was unclear because the effect of abemaciclib was dominant in reducing CDK4 and cyclin D1 levels. Thus, the combination treatment with the CDK4 inhibitors abemaciclib and desipramine was more prominent in HCT-116 cancer cells than in H460 or MCF7 cells (Figure 2B). Based on these results, we evaluated the synergistic effects of abemaciclib and desipramine in HCT-116 cells.

### 2.2. Enhanced Effects of Abemaciclib and Desipramine Combination on Cell Cycle and Death

**Figure 3:**
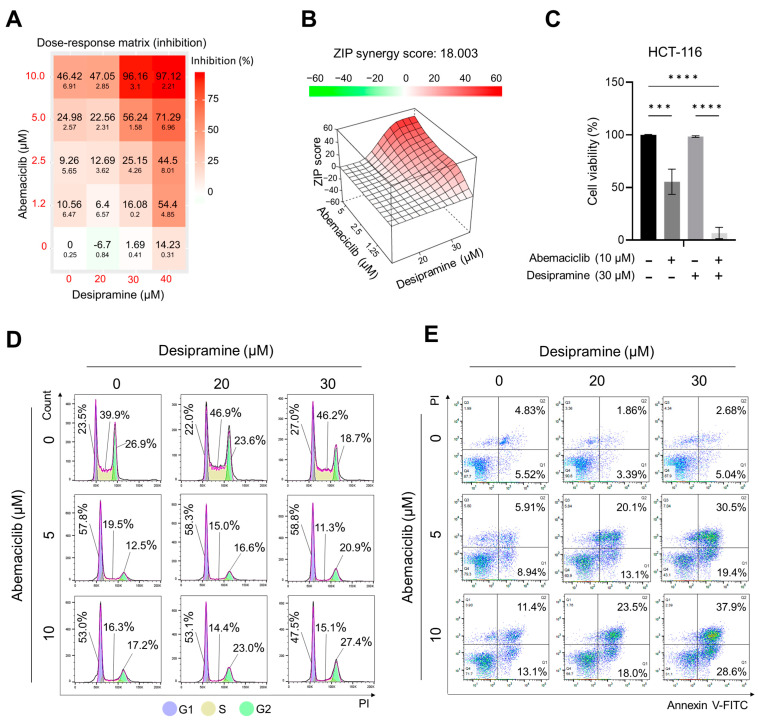
Synergistic effect of abemaciclib and desipramine combination treatment on HCT-116 cells. (**A**) Dose–response matrix of inhibition rate (%) ± SEM (n = 3) of combination treatment between abemaciclib and desipramine on HCT-116 cells visualized by analysis using SynergyFinder 3.0. (**B**) Mapping of synergistic effect of abemaciclib and desipramine combination treatment on HCT-116 cells based on ZIP scores calculated using SynergyFinder 3.0. (**C**) Relative cell viability for the maximal synergistic concentration of HCT-116 cells with combination treatment. Data are presented as means ± standard errors of the mean, and *p*-values (n = 3) were determined by one-way analysis of variance, followed by a Tukey multiple comparison test (*** *p* < 0.001; **** *p* < 0.0001). (**D**) Cell cycle analyses of HCT-116 cells treated with abemaciclib and/or desipramine for 24 h. (**E**) Annexin V-FITC/propidium iodide (PI) dual staining of HCT-116 after treatment with abemaciclib and/or desipramine for 72 h.

To evaluate the synergistic effect of abemaciclib and desipramine, we analyzed the viability of HCT-116 cells with combinations of four concentrations of abemaciclib (1.25, 2.5, 5, and 10 μM) and three concentrations of desipramine (20, 30, and 40 μM). Hence, the maximum inhibitory effect on cell viability (97.12%) was observed with the combination of 10 μM abemaciclib and 40 μM desipramine (Figure 3A). Meanwhile, when the synergistic effect of abemaciclib and desipramine was assessed, the highest synergistic effect (ZIP score: 18.003) was observed with the combination of 10 μM abemaciclib and 30 μM desipramine (Figure 3B). Repeated CellTiter-Glo assays (n = 3) confirmed a significant reduction in relative cell viability by 96.16 ± 3.10% with the combination of abemaciclib (10 μM) and desipramine (30 μM) compared to single-drug treatments of either 10 μM abemaciclib (46.42 ± 6.91%, *p* < 0.001) or 30 μM desipramine (1.69 ± 0.41%, N.S.) alone (Figure 3C).

To determine whether the combination treatment inhibited cellular proliferation, we analyzed cell cycles. Single treatment with abemaciclib increased the G1 population in HCT-116 cells, as the concentration was increased from G1-23.5% at a negative control to 57.8% at 5 μM and 53.0% at 10 μM. By contrast, a higher desipramine concentration did not increase the G1 population at 20 μM (22.0%) or 30 μM (27.0%). For the G2 population, abemaciclib decreased the G2 level from 26.9% at a negative control to 12.5% at 5 μM and to 17.2% at 10 μM. Desipramine also reduced the G2 population to 23.6% at 20 μM and to 18.7% at 30 μM. The S-phase population treated with abemaciclib was reduced from 39.9% at a negative control to 19.5% at 5 μM and 16.3% at 10 μM, and single treatment with desipramine was not effective for S-phase reduction even at higher concentrations. However, we confirmed that the G1 arrest was increased to the highest level (58.8%) with the combination of abemaciclib (5 μM)/desipramine (30 μM), and G2 arrest was increased to the highest level at 27.4% with the abemaciclib (10 μM)/desipramine (30 μM) combination. Meanwhile, the S-phase level decreased to 11.3% with the abemaciclib (5 μM)/desipramine (30 μM) combination (Figure 3D). Therefore, the combination of lower abemaciclib concentrations is sufficiently effective in inducing G1- and G2-phase arrests and depleting the S phase.

To compare the effect of abemaciclib/desipramine combination treatment and single-drug treatment on cell death, we analyzed the changes in both early and late apoptotic populations by Annexin-V assay using flow cytometry. The single abemaciclib treatment increased the frequency of the early and late apoptotic population dose-dependently, from early (5.52%) and late (4.83%) apoptotic population of negative control HCT-116 cell to 13.1% early and 11.4% late apoptotic population frequencies at 10 μM. Unlike abemaciclib, desipramine did not dose-dependently increase the apoptotic population at 20 μM (3.39% in early and 1.86% in late apoptotic populations) and at 30 μM (5.04% in early and 2.68% in late apoptotic populations). However, applying those two drugs in combination significantly increased the apoptotic population (33.2% in total; 13.1% in early and 20.1% in late apoptotic populations) even at the lowest concentration of the abemaciclib (5 μM)/desipramine (20 μM) combination. The highest level of overall cell death at 66.5% (28.6% in early and 37.9% in late apoptotic populations) was observed with the combination of higher abemaciclib (10 μM)/desipramine (30 μM) concentrations. However, the combination of lower abemaciclib (5 μM) with a higher concentration of desipramine (30 μM) also resulted in a 49.9% of apoptotic population (19.4% in early and 30.5% in late apoptotic populations) (Figure 3E). Desipramine did not induce cell death by itself, even at higher doses; however, when used in combination with abemaciclib, it enhanced the potential cytotoxic effect of abemaciclib at lower concentrations, suggesting that abemaciclib can be used at a lower dose with minimal adverse effects when applied in combination with desipramine. This result provides a basis for assessing the effectiveness of the combined use of abemaciclib/desipramine in vivo.

### 2.3. Antitumor Effects of Abamaciclib and Desipramine in an HCT-116 Xenograft Model

**Figure 4:**
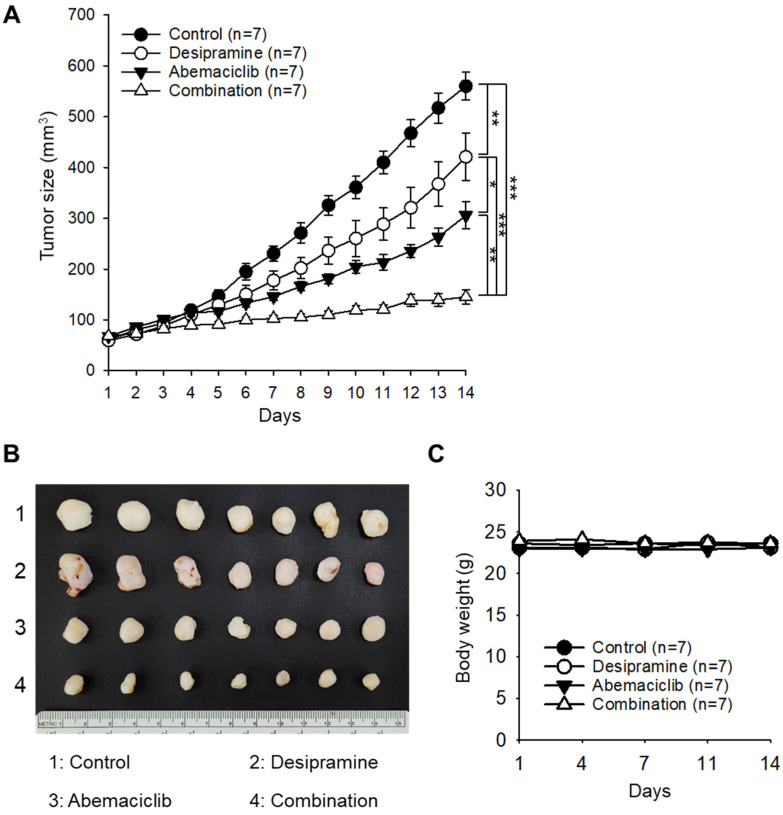
Therapeutic effect of the combined treatment of abemaciclib and desipramine in HCT-116 xenograft colon tumor model. (**A**) Changes in tumor sizes over time measured for each treatment group. Significant difference (*p*-values) was calculated using a one-way analysis of variance (* *p* < 0.1, ** *p* < 0.01, and *** *p* < 0.001). (**B**) Photograph of tumors collected from the mice on day 14. (**C**) Changes in body weights of mice over time recorded in each treatment group.

To evaluate the enhanced therapeutic effect of the combined treatment with abemaciclib and desipramine, a xenograft tumor model was constructed using the colorectal cancer cell line HCT-116. When the tumor size reached 60–70 mm^3^, the drugs were administered once daily at 20 mg/kg (desipramine) and/or 30 mg/kg (abemaciclib). Tumor sizes of the desipramine- or abemaciclib-treated groups were 75.2% (*p* < 0.01) and 54.6% (*p* < 0.001) of the control group, respectively. Notably, tumor size in the combined treatment group significantly decreased to 25.9% (*p* < 0.001) compared with that in the control group, confirming a significant synergistic antitumor effect (coefficient of drug interaction (CDI) = 0.64) in the combined treatment group compared with the drug-alone treatment groups (Figure 4A,B). The mice in the drug treatment groups did not show a significant difference in body weight compared with the control mice (Figure 4C). In an additional set of experiments, we found that the anticancer effect of the combination treatment (desipramine 20 mg/kg + abemaciclib 30 mg/kg) was similar to that of high-dose abemaciclib (50 mg/kg) (Figure S1). The combination of desipramine (20 mg/kg) and high-dose abemaciclib (50 mg/kg) did not induce an enhanced antitumor effect compared with either a high dose of abemaciclib (50 mg/kg) or combined treatment with a lower dose of abemaciclib (30 mg/kg).

**Figure 5:**
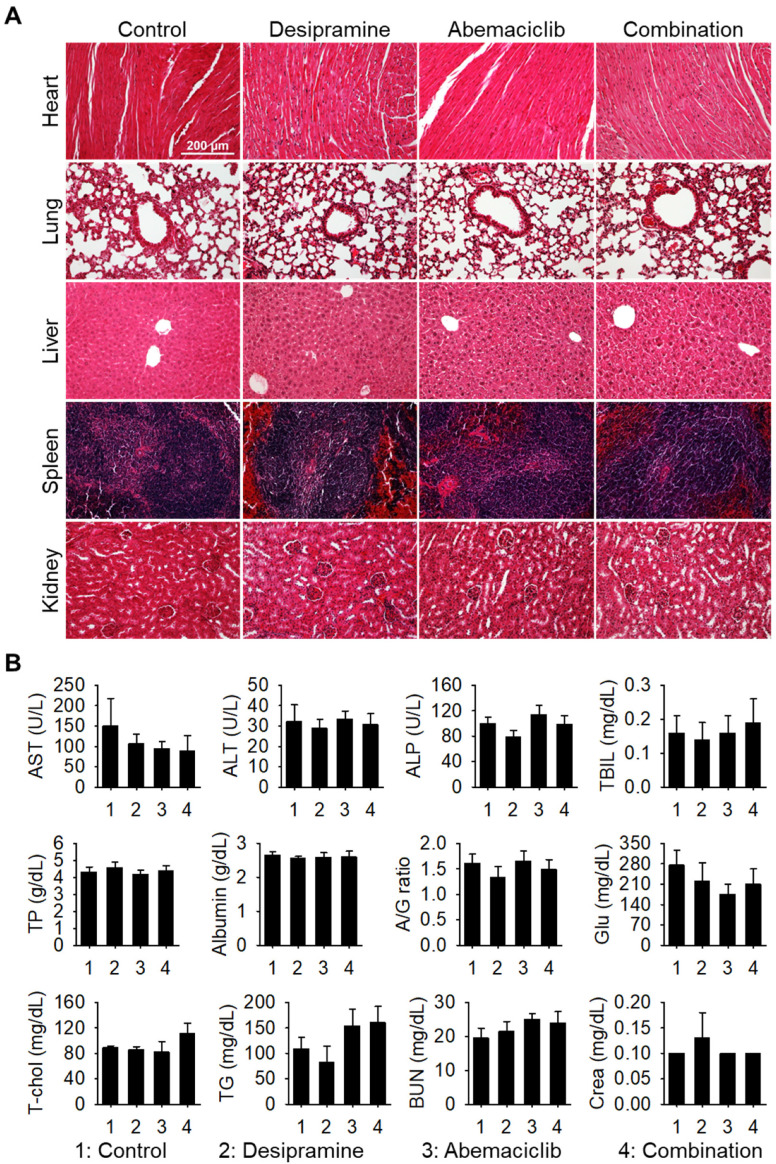
Evaluation the drug toxicity in vivo. (**A**) Hematoxylin and eosin-stained sections of the heart, lung, liver, spleen, and kidney of the mice were obtained after 14 days. (**B**) Data of serum biochemical analysis of the mice on day 14. There were no statistically significant differences between any of the groups and the control group. AST, aspartate transaminase; ALT, alanine transaminase; ALP, alkaline phosphatase; TBIL, total bilirubin; TP, total protein; A/G ratio, albumin/globulin ratio; Glu, glucose; T-chol, total cholesterol; TG, triglyceride; BUN, blood urea nitrogen.

Next, we performed histopathological analysis of hematoxylin and eosin (H&E)-stained sections of the major organs (heart, lung, liver, spleen, and kidney). No histological changes were observed in the drug-treated groups compared with the vehicle-treated control group (Figure 5A). In addition, no signs of side effects were observed in the blood chemical analysis (Figure 5B), confirming that the combined dosage regimen used in this study is safe and provides synergistic antitumor effects against colon tumors.

Therefore, the combination therapy of CDK4/6 inhibitors with antidepressants could be a novel therapeutic approach for solid tumors such as colorectal, breast, and lung tumors.

## 3. Discussion

Assuming that cell cycle progression is hyperactive in almost all cancers, CDKs are crucial for the transition from the G1 to S phase of the cell cycle. Thus, CDK4/6 inhibitors have been recommended as promising targeted therapeutics for various cancers. The three main CDK4/6 inhibitors that are currently approved and widely studied are palbociclib, ribociclib, and abemaciclib. Each inhibitor has unique properties and varying degrees of relevance for different cancer types; however, their main indication remains HR(+)/HER2(−) breast cancer [24]. Comparisons of their efficacy based on clinical trials revealed that abemaciclib showed broader potential relevance beyond breast cancer, including NSCLC and glioblastoma; however, in terms of objective response rate or progression-free survival, it is less compelling than breast cancer [25]. This has led to extensive studies on the combination of various small-molecule drugs, including epidermal growth factor receptor inhibitors in esophageal squamous cell carcinoma, BRAF or MEK inhibitors in melanoma [26,27], ALK inhibitors in neuroblastoma [28], and immune checkpoint inhibitors [29]. To utilize this combination therapy, large-scale clinical trials are required to evaluate the toxicity that causes serious side effects, even if the combination therapy is more efficient. Therefore, drugs that can overcome this obstacle and be safely used in combination with CDK4/6 inhibitors need to be identified.

Herein, we propose the use of a TCA, desipramine, as a combination drug to enhance the therapeutic outcomes of CDK4/6 inhibitors. It has been observed that the prescription of antidepressants, widely used among cancer patients to treat neuropathic pain, reduces the risk of mortality in cancer patients [30]. Particularly, TCAs have been reported to be more effective in reducing mortality compared to other types of antidepressants [15,17]. Recent studies have explored the mechanisms of various antidepressants to modulate autophagy, a process involved in the degradation and recycling of cellular components that contribute to their anticancer effects. These antidepressants may trigger apoptosis, inhibit cellular energy metabolism, and exhibit other mechanisms that suppress tumor growth [31]. Previous reports have shown that when desipramine is administered to liver cancer cell lines, the activity of proteins such as p38 and ERK, which are involved in the pro-apoptotic signaling pathway, rapidly increases, supporting its potential as a cancer therapeutic [32].

In this study, desipramine treatment alone in HCT-116 cells reduced the protein levels of CDK4 and cyclin D1, although it did not increase PARP cleavage (Figure 2B) or did not increase the apoptotic cell population, even at higher concentrations (Figure 3E). However, when combined with abemaciclib, the frequency of apoptosis dramatically increased to more than twice that of abemaciclib treatment alone (Figure 3E). The arrest of cell cycle at G2 increased by 10% with the combination of desipramine and abemaciclib, regardless of abemaciclib dose difference. Therefore, the non-cytotoxicity of desipramine may be attributed to the huge increase in apoptotic cell frequency with a lower dose of abemaciclib, suggesting that this combination avoids severe adverse effects on non-cancerous cells by using a low dose of abemaciclib.

These results were also verified in vivo by comparing tumor growth inhibition with drug treatment without toxicity to non-tumor organs or blood biochemistry. The combination of desipramine (20 mg/kg) and a lower dose of abemaciclib (30 mg/kg) resulted in a significantly synergistic antitumor effect when compared with treatment with each drug alone, whereas no adverse effect was observed in the combined treatment group. Interestingly, the combined treatment with desipramine and high-dose abemaciclib (50 mg/kg) did not induce further antitumor effects. This is in good agreement with the results obtained from the in vitro cell study and suggests an optimal dose regimen for combination therapy.

Although combining CDK4/6 inhibitors with other targeted therapies can enhance the efficacy of cancer treatment, it also tends to increase the risk of adverse effects. This necessitates a careful and personalized approach to treatment, guided by ongoing research and clinical trials, to optimize therapeutic strategies. Our study on the combination of abemaciclib and desipramine may contribute to these efforts.

## 4. Materials and Methods

### 4.1. Cell Culture

H460, MCF7, and HCT-116 cells were purchased from American Type Culture Collections (Manassas, VA, USA). H460 and MCF7 were cultured in RPMI-1640 medium (#10-040-CV; Corning, New York, NY, USA) supplemented with 10% (*v*/*v*) fetal bovine serum (#35-010-CV; Corning) and 1% (*v*/*v*) penicillin–streptomycin antibiotic cocktail (#15240062; Thermo Fisher Scientific, Waltham, MA, USA), whereas HCT-116 was cultured in McCoy’s 5A medium supplemented with 10% (*v*/*v*) fetal bovine serum and 1% (*v*/*v*) penicillin–streptomycin in a humidified atmosphere at 37 °C and 5% CO_2_. All cells were sub-cultured at 80–90% confluence twice weekly, detached with TrypLE Express solution (#25200072, Thermo Fisher Scientific, Waltham, MA, USA) to minimize the damage to the cells, and maintained in the absence of mycoplasma contamination.

### 4.2. Cell Viability Assay

To measure the effects of desipramine hydrochloride (#D3900; Sigma-Aldrich, St. Louis, MO, USA) and abemaciclib (#S5746; Selleckchem, Houston, TX, USA) in vitro, H460, MCF7, and HCT-116 cells were seeded at a density of 3 × 10^3^ cells/well in 96-well white optical plates (#3903; Corning, New York, NY, USA) and then treated with 8-point serial half-log dilutions of desipramine (~200 μM) or abemaciclib (~200 μM) from the next day for 72 h. Cell viability was measured using CellTiter-Glo (#G9241; Promega, Madison, WI, USA) according to the manufacturer’s protocol on a Synergy HTX multimode plate reader (Agilent BioTek, Santa Clara, CA, USA) and normalized to that of the negative control. The IC_50_ for each drug and cell line was analyzed using GraphPad Prism (ver. 9; GraphPad Software, Boston, MA, USA).

### 4.3. Combinatorial Cytotoxicity Assay

To evaluate the combinatorial effects of abemaciclib and desipramine, the HCT-116 cell was selected, and combined compounds of abemaciclib (0–10 μM) and desipramine (0–30 μM) were treated for 72 h at 37 °C in a 5% CO_2_ incubator. Cell viability was measured as described above, and synergistic scores (ZIP) were calculated using the SynergyFinder 3.0 software (version 3.0) [33].

### 4.4. Immunoblotting

Protein concentrations were measured using a bicinchoninic acid kit (#23225; Thermo Fisher Scientific, Carlsbad, CA, USA). Proteins were separated by sodium dodecyl sulfate-polyacrylamide gel electrophoresis and transferred onto Immobilon-P membrane (#IPVH00010; Merck, Burlington, MA, USA). Primary antibodies against PARP (#9542S; Cell Signaling Technology, Beverly, MA, USA), CDK4 (#12790S; CST), Cyclin D1 (#2978S; CST), procaspase-3 (#sc-7272; Santa Cruz Biotechnology, Dallas, TX, USA), and glyceraldehyde-3-phosphate dehydrogenase (#sc-25778; Santa Cruz Biotechnology) were added and incubated overnight at 4 °C. Horseradish peroxidase-conjugated secondary antibodies specific to the primary antibodies were used to detect proteins using a chemiluminescence kit (#34577; Thermo Fisher Scientific, Carlsbad, CA, USA).

### 4.5. Cell Cycle Analysis and Apoptosis Assay Using Flow Cytometry

Compound treated cells, including a dimethylsulfoxide (DMSO; #D8418; Sigma-Aldrich) control, were prepared fixed in cold 70% ethanol for 16 h at 4 °C in 300 μL of FxCycle™ propidium iodide/RNase staining solution (#F10797; Thermo Fisher Scientific, Invitrogen, Carlsbad, CA, USA) and then transferred into a 5 mL round-bottom polystyrene tube with a cell-strainer cap (#352235; Corning, Falcon, México, S.A de C.V) after incubation for 30 min at room temperature. The cell cycle was analyzed using a FACSLyric™ Flow Cytometer (BD Biosciences, Santa Clara, CA, USA).

To measure apoptotic populations, whole cells, including adherent and floating cells, were collected using the FITC Annexin V Apoptosis Detection Kit (#556547; BD Biosciences) according to the manufacturer’s protocol. Briefly, HCT-116 (1.5 × 10^5^) cells were treated with combination of abemaciclib (10 μM) and desipramine (20 or 30 μM) for 72 h in a 6-well plate. Harvested cells were stained for 15 min at room temperature in the dark, and the stained cells were analyzed using the FACSLyric™ Flow Cytometer. All flow cytometry data were analyzed using the FlowJo software (version 10; FlowJo LLC, Ashland, OR, USA).

### 4.6. Xenograft Tumor Model

HCT-116 cancer cells were implanted subcutaneously into BABL/c nude mice (CAnN.CgFoxn1nu/CrljOri; Orient Bio, Seoul, Republic of Korea) at 5 × 10^6^ cells/100 μL. When the tumor size reached 60–70 mm^3^, the mice were randomly divided into four groups as follows: control (n = 7), desipramine alone (n = 7), abemaciclib alone (n = 7), and desipramine and abemaciclib combination (n = 7). Desipramine (20 mg/kg) was injected intraperitoneally, whereas abemaciclib was administered orally at a dose of 30 mg/kg. Desipramine and abemaciclib were administered once daily until the end of the study period. The vehicle was administered to mice in the control group. Tumor size was measured every day until day 14 after treatment, and the differences between the groups were analyzed. Tumor size was calculated as follows: tumor volume (mm^3^) = 1/2 × length (mm) × width (mm) × height (mm). The body weights of the mice were measured periodically. The in vivo synergistic effect of the combined treatment was determined by calculating the CDI [34]. CDI values of <1 and <0.7 indicate synergy and significant synergy, respectively. The clinically recommended dose range for desipramine is 50–300 mg per day [35]. Desipramine was administered to the mice at a dose of 20 mg/kg/day to evaluate its antidepressant effect [36]. We also tested this drug at the same dose in an antitumor study.

To evaluate the in vivo toxicity of the drugs, major organs (heart, lung, liver, spleen, and kidney) were collected on day 14. The tissues were fixed in 10% formalin, embedded in paraffin, and sectioned into 4 μm thick slices. These sections were subsequently stained with H&E using an automated system. Blood samples were also collected from the mice on day 14 for serum biochemical analysis.

### 4.7. Statistical Analysis

All data except blood chemistry data are expressed as mean ± SEM, while blood chemistry data are expressed as mean ± SD. A one-way ANOVA test was carried out to analyze the significant differences between test groups (* *p* < 0.05, ** *p* < 0.01, and *** *p* < 0.001). The in vitro and in vivo data were analyzed using one-way ANOVA, followed by Tukey’s multiple comparison test and Holm–Sidak multiple comparison test, respectively.

## 5. Conclusions

The combined treatment of desipramine, a tricyclic antidepressant known to significantly improve survival rates in cancer patients, and abemaciclib, a CDK4/6 inhibitor applicable to various cancer types, induces a significantly synergistic effect in colorectal cancer therapy without noticeable side effects from abemaciclib. As mentioned above, CDK4/6 inhibitors are mostly applied to patients with HR+ breast cancer. Here, we tested the therapeutic efficacy of abemaciclib and desipramine combination in lung, TNBC, and colon cancer cell lines to explore the potential for expanding this combination regimen to other types of cancers with high incidence or mortality. In this study, we demonstrated for the first time the synergistic effect of a CDK4/6 inhibitor and TCA combination. We are currently conducting research to elucidate the detailed mechanism of the combination.

Although further in vivo studies, including dose-dependent toxicity and survival rate analyses, are necessary, this study suggests that the combination therapy of CDK4/6 inhibitors and antidepressants may represent a novel therapeutic approach for solid tumors such as colorectal, breast, and lung cancers.

## Data Availability

The data that support the findings of this study are available from the corresponding author upon reasonable request. Some data may not be made available because of privacy or ethical restrictions.

## Supplementary Materials

The following supporting information can be downloaded at: https://www.mdpi.com/article/10.3390/ijms25137407/s1.
