# Supplementary material for: Synergistic Enhancement of Antitumor Effects by Combining Abemaciclib with Desipramine
Source: Int J Mol Sci. 2024 Jul 5;25(13):7407. doi: 10.3390/ijms25137407 (PMC11242104; doi:10.3390/ijms25137407)
Supplement: Supplementary file 1 [file ijms-25-07407-s001.zip › ijms-3068185-supplementary.pdf]

Supplementary Information

Synergistic Enhancement of Antitumor Effects by Combining Abemaciclib with Desipramine

Yan Li [1,†], Yeojin Sung [2,†] , Young Eun Choi [2] , Yongdoo Choi [1,*] and Sung-Ho Goh [2,*]

[1]Division of Technology Convergence, National Cancer Center, 323 Ilsan-ro, Goyang, Gyeonggi-Do 10408, Republic of Korea.

[2]Division of Cancer Biology, Research Institute and Hospital, National Cancer Center, 323 Ilsan-ro, Goyang, Gyeonggi-Do 10408, Republic of Korea.

[†]These authors contributed equally to this study

*Correspondence: ydchoi@ncc.re.kr (Y.C.), andrea@ncc.re.kr (S.H.G)

1. Antitumor effects of abamaciclib and desipramine in HCT-116 xenograft model

HCT-116 cancer cells were implanted subcutaneously into BABL/c nude the mice (CAnN.CgFoxn1nu/CrljOri; Orient Bio, Seoul, Korea) at 5×10^6 cells/100 μl, forming tumors. When tumor size reached 60-70 mm^3, the mice were randomly divided into 4 groups: a control group (n = 7), a vehicle control group (n = 7), a desipramine alone treatment group (n = 7), an abemaciclib alone treatment group (30 mg/kg, n = 7), an abemaciclib alone treatment group (50 mg/kg, n = 7), a combined treatment group with desipramine (20 mg/kg) and abemaciclib (30 mg/kg) (n = 7), and a combined treatment group with desipramine (20 mg/kg) and abemaciclib (50 mg/kg) (n = 7). Desipramine was administered intraperitoneally at a dose of 20 mg/kg, while abemaciclib was given orally at doses of either 30 or 50 mg/kg. Both drugs were administered once daily until the end of the experiment. Then, the tumor size was measured daily until day 14 post-treatment, and the differences between each group were analyzed. The tumor size was calculated as follows: Tumor volume (mm^3) = 1/2 × length (mm) × width (mm) × height (mm). Body weight of the mice was also measured periodically.

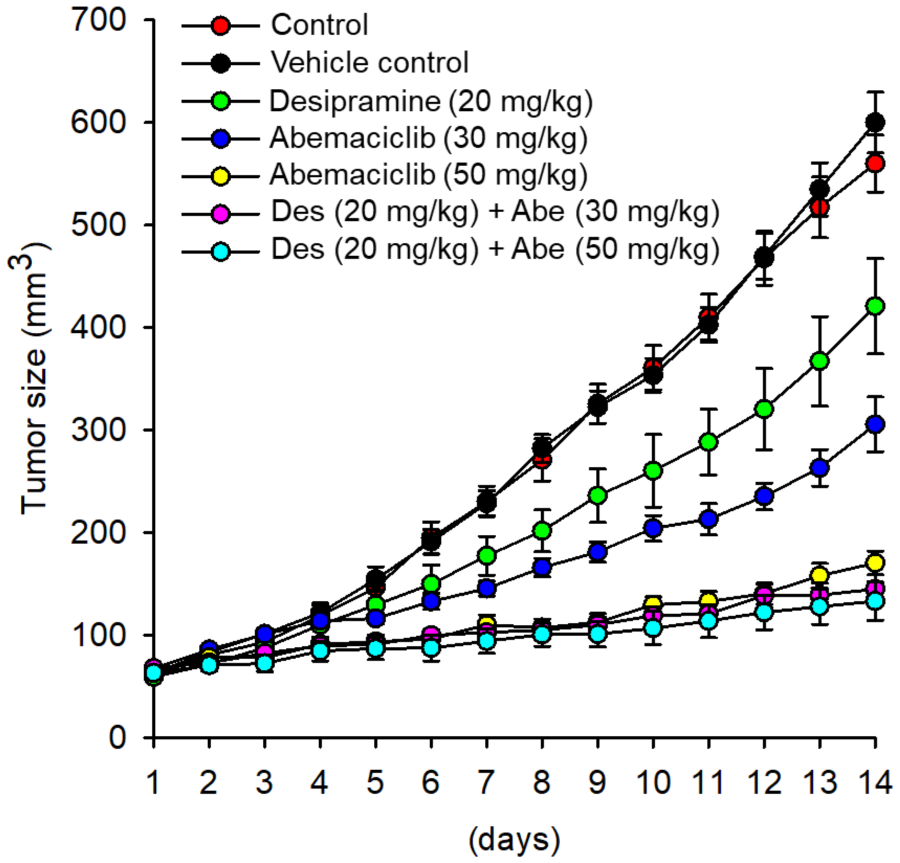

Figure S1 Changes in tumor sizes over time were measured for each treatment group. No significant differences were observed between the control and vehicle control groups. Additionally, no significant differences were found among the following groups: abemaciclib alone (50 mg/kg), the combination of desipramine (20 mg/kg) and abemaciclib (30 mg/kg), and the combination of desipramine (20 mg/kg) and abemaciclib (50 mg/kg).
